# Supplementary material for: Relationship between weekends catch-up sleep and risk of aging
Source: PLoS One. 2025 Oct 8;20(10):e0332584. doi: 10.1371/journal.pone.0332584 (PMC12507208; doi:10.1371/journal.pone.0332584)
Supplement: S1 File — (DOCX) [file pone.0332584.s001.docx]

**Supplementary materials**

Supplementary Method 1

Code for calculating phenotypic age:

install.packages("devtools")

devtools::install_github("dayoonkwon/BioAge")

library(BioAge) #topic of example

library(dplyr)

#HD using NHANES (separate training for men and women)

hd = hd_nhanes(biomarkers=c("albumin","alp","lncrp","totchol","lncreat","hba1c","sbp","bun","uap","lymph","mcv","wbc"))

#KDM bioage using NHANES (separate training for men and women)

kdm = kdm_nhanes(biomarkers=c("albumin","alp","lncrp","totchol","lncreat","hba1c","sbp","bun","uap","lymph","mcv","wbc"))

#phenoage using NHANES

phenoage = phenoage_nhanes(biomarkers=c("albumin_gL","alp","lncrp","totchol","lncreat_umol","hba1c","sbp","bun","uap","lymph","mcv","wbc"))

#assemble NHANES IV dataset with projected biological aging measures for analysis

data = merge(hd$data, kdm$data) %>% merge(., phenoage$data)

Supplementary Method 2

In this study, the selection of adjusted variables was guided by theoretical frameworks, prior literature on aging and sleep research, and potential confounding pathways.

Chronological age
Chronological age was included as an adjustment variable despite phenotypic age (biological aging) being the primary outcome, as the two constructs capture distinct aspects of aging. Chronological age reflects the passage of time, while phenotypic age quantifies biological aging processes that may diverge from chronological progression (e.g., individuals with the same chronological age can exhibit different rates of biological aging). By adjusting for chronological age, we aimed to isolate the association between CUS and accelerated biological aging (i.e., phenotypic age relative to chronological age), rather than conflating it with age-related changes tied to time alone. This approach aligns with standard practices in aging research to disentangle biological from chronological aging [1].

Body Mass Index (BMI)
BMI was included as a covariate due to its well-established link to biological aging pathways, including chronic inflammation, metabolic dysfunction, and oxidative stress—all of which are hypothesized to drive aging processes [2]. Even if BMI was not significantly associated with the outcome in our dataset, it may act as a confounder by potentially influencing both sleep behaviors (e.g., CUS patterns) and biological aging. Controlling for BMI helps minimize residual confounding and strengthens the validity of the observed association between CUS and phenotypic age.

Educational level
Educational attainment was adjusted for as a key social determinant of health, as it correlates with health literacy, access to healthcare, and lifestyle behaviors (e.g., sleep hygiene, stress management) that may independently affect aging trajectories [3,4]. Prior studies have shown that education influences both sleep patterns and biological aging, making it a potential confounder in the relationship between CUS and phenotypic age. Inclusion of this variable helps account for socioeconomic disparities that could bias the association.

Marital status
Marital status was included based on evidence that relationship status affects health behaviors, stress levels, and social support—factors known to impact sleep quality and aging. Married individuals may have more regular sleep schedules or better access to health resources, while unmarried individuals may experience higher stress that accelerates biological aging. Adjusting for marital status helps control for these social contextual factors that could confound the link between CUS and phenotypic age [5].

In summary, all adjusted variables were selected a priori to address potential confounding, enhance internal validity, and align with methodological precedents in the field, rather than being driven solely by statistical significance in our dataset.

1. Levine ME. Modeling the rate of senescence: can estimated biological age predict mortality more accurately than chronological age? J Gerontol A Biol Sci Med Sci. 2013 Jun;68(6):667-74. doi: 10.1093/gerona/gls233. Epub 2012 Dec 3. PMID: 23213031; PMCID: PMC3660119.
2. Santos AL, Sinha S. Obesity and aging: Molecular mechanisms and therapeutic approaches. Ageing Res Rev. 2021 May;67:101268. doi: 10.1016/j.arr.2021.101268. Epub 2021 Feb 5. PMID: 33556548.
3. Zahodne LB, Zajacova A. Education and Cognitive Aging: An Introduction to the Special Section. J Gerontol B Psychol Sci Soc Sci. 2020 Aug 13;75(7):e78-e80. doi: 10.1093/geronb/gbaa091. PMID: 32716028.
4. Lleras-Muney A, Price J, Yue D. The association between educational attainment and longevity using individual-level data from the 1940 census. J Health Econ. 2022 Jul;84:102649. doi: 10.1016/j.jhealeco.2022.102649. Epub 2022 Jun 27. PMID: 35793610.
5. August KJ. Marital Status, Marital Transitions, and Sleep Quality in Mid to Late Life. Res Aging. 2022 Mar-Apr;44(3-4):301-311. doi: 10.1177/01640275211027281. Epub 2021 Jun 28. PMID: 34180305.

Figure S1 Sleep duration among different ages


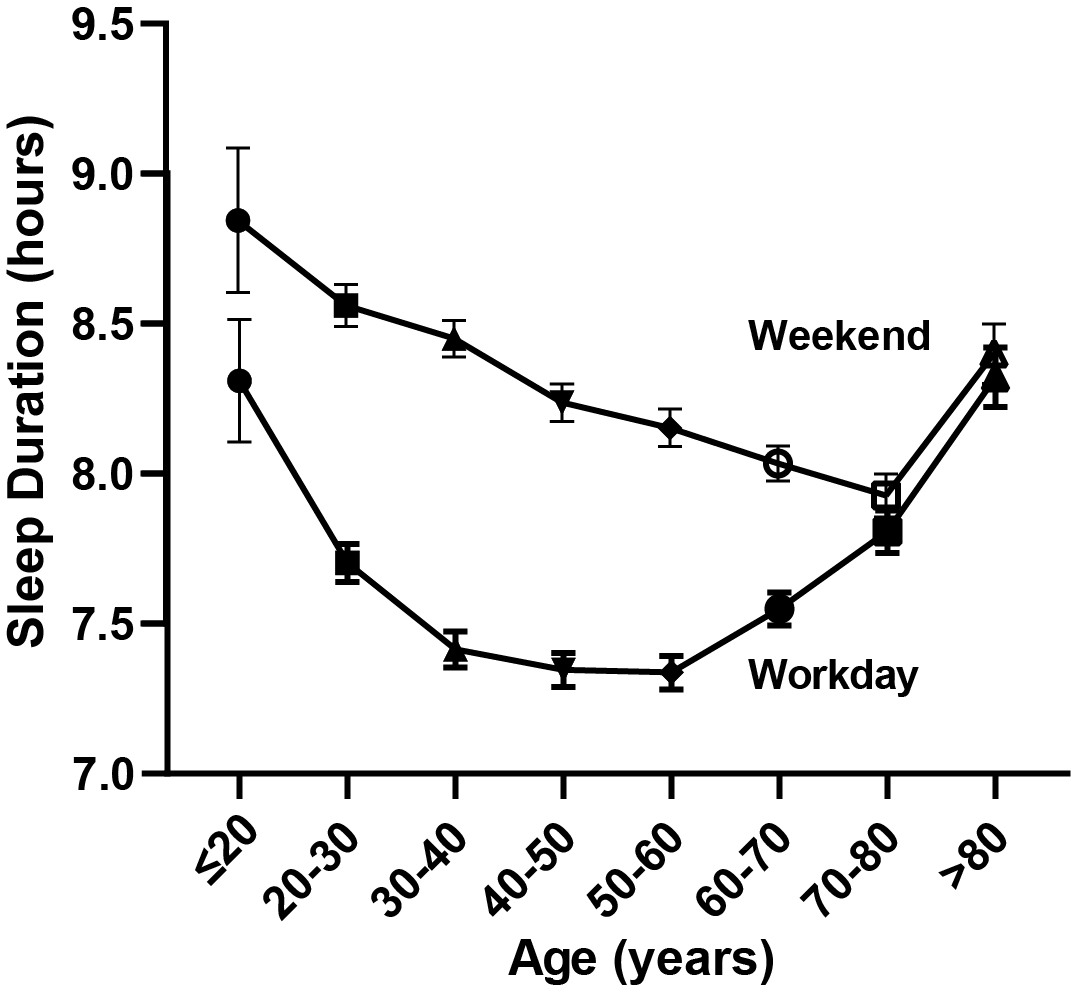


Figure S2 The nonlinear relationship between weekend CUS and aging risk


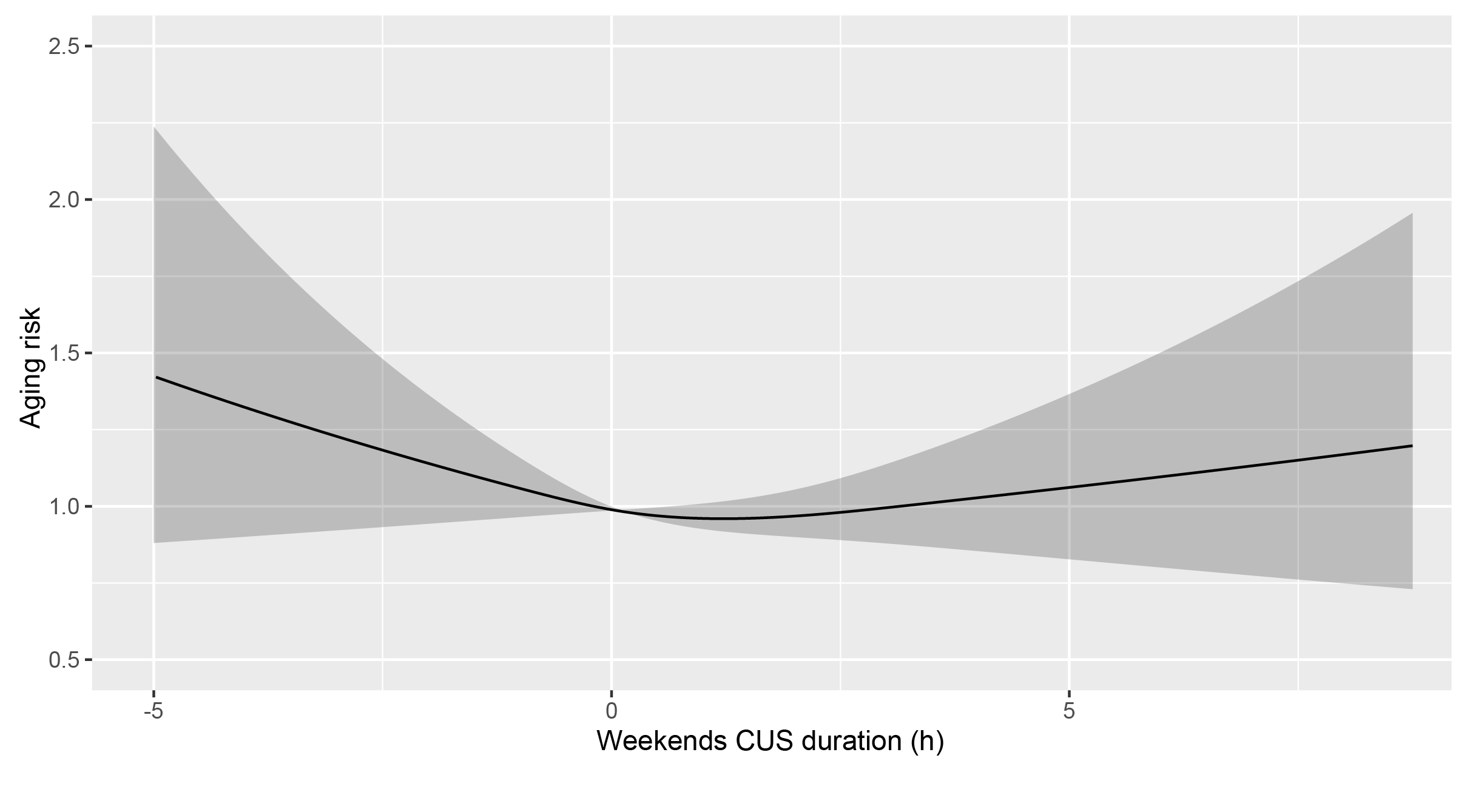


Figure S3 The relationship between CUS and indicators related to cognitive aging.


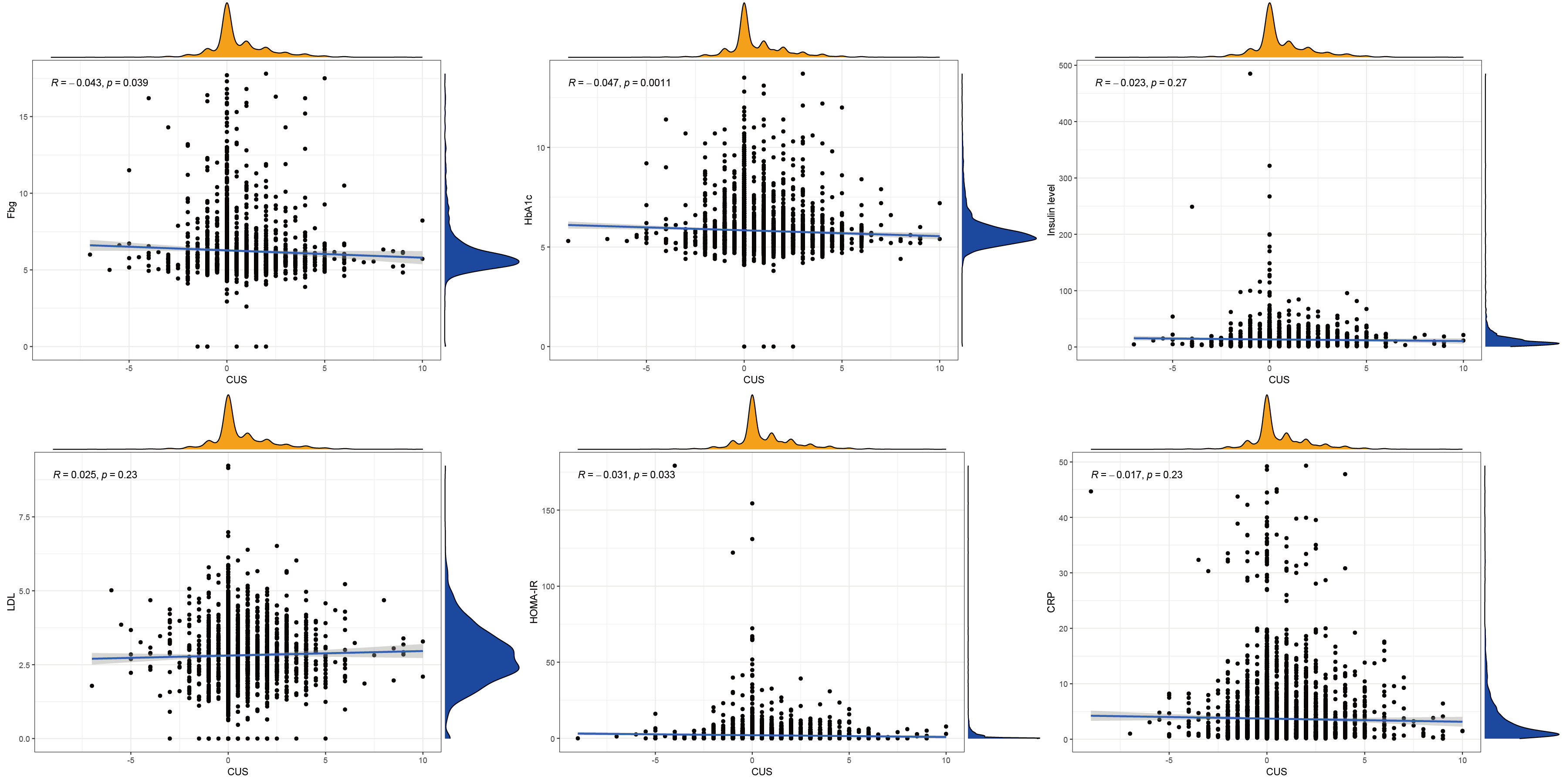


Figure S4 The nonlinear relationship between sleep duration and aging risk


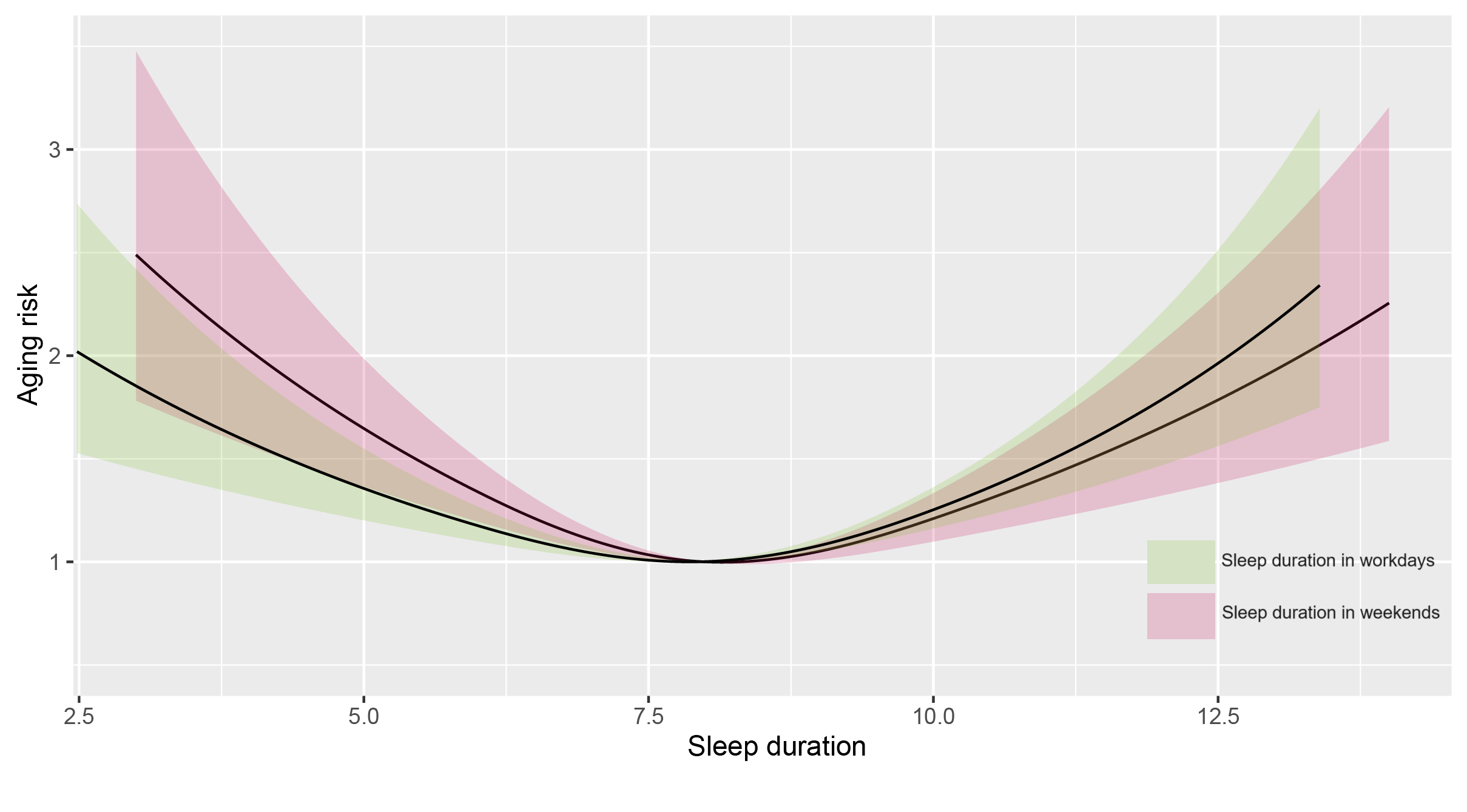


Figure S5 Mediation Analyses


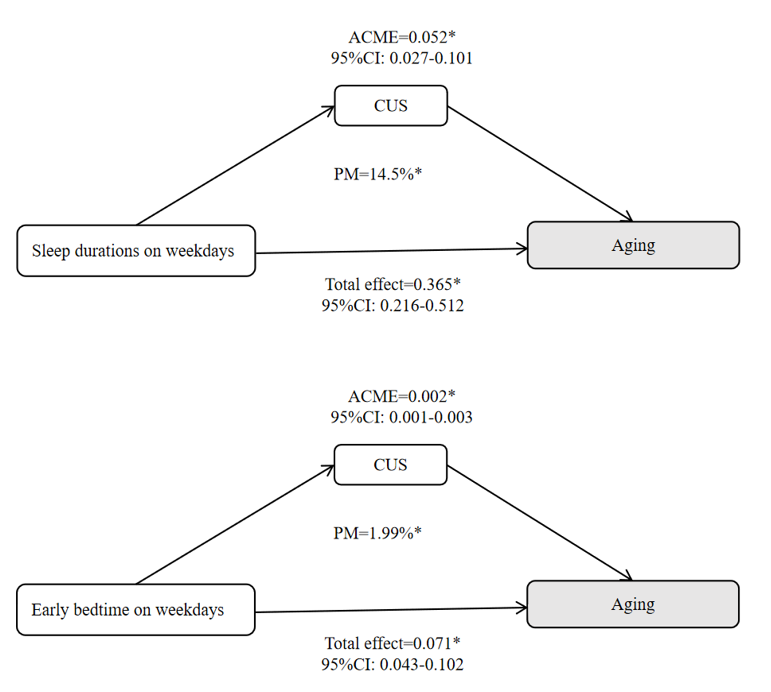


Table S1 The relationship between bedtime and aging risk

| Bedtime | Model 1 | | Model 2 | | Model 3 | |
| --- | --- | --- | --- | --- | --- | --- |
| Before 0:00 at workday | Ref. |  | Ref. |  | Ref. |  |
| After 0:00 at workday | **1.47 (1.27, 1.69)** | **<0.001** | **1.42 (1.17, 1.73)** | **0.003** | **1.41 (1.11, 1.79)** | **0.017** |
| Before 0:00 at weekends | Ref. |  | Ref. |  | Ref. |  |
| After 0:00 at weekends | **1.26(1.09, 1.45)** | **0.004** | **1.23(1.04, 1.46)** | **0.022** | **1.21(1.01, 1.45)** | **0.040** |

Model 1 was crude model.

Model 2 was adjusted age, sex, marital status, PIR, educational level, obesity

Model was adjusted age, sex, marital status, PIR, educational level, obesity, smoke, alcohol use, sleep trouble, PA, sleep duration in work day.

Table S2 The relationship between CUS and aging risk at different bedtime in weekends

| Before 0:00 at weekends |  |  | After 0:00 at weekends |  | P for interaction |
| --- | --- | --- | --- | --- | --- |
| Weekend CUS |  | P |  | P | 0.448 |
| No | Ref. |  | Ref. |  |  |
| Yes | 0.77 (0.53, 1.12) | 0.144 | 0.80 (0.57, 1.12) | 0.163 |  |
| Weekend CUS duration (h) |  |  |  |  | 0.842 |
| ≤0 | Ref. |  | Ref. |  |  |
| 0-1* | **0.65(0.45, 0.95)** | **0.032** | 0.87(0.60, 1.28) | 0.399 |  |
| 1-2* | 0.73(0.50, 1.06) | 0.084 | 0.72(0.42, 1.22) | 0.170 |  |
| >2 | 1.07(0.53, 2.19) | 0.810 | 0.81(0.48, 1.37) | 0.356 |  |

*0h<Weekend CUS duration≤1h, 1h<Weekend CUS duration≤2h.

Model was adjusted age, sex, marital status, PIR, educational level, obesity, smoke, alcohol use, sleep trouble, PA, sleep duration in work day.

Table S3 The relationship between sleep duration and aging

|  | Sleep duration  in workdays |  | Sleep duration in  weekends |  | P for interaction |
| --- | --- | --- | --- | --- | --- |
|  | OR (95%CI) | P | OR (95%CI) | P | 0.026 |
| 7-8 hours | Ref. |  | Ref. |  |  |
| < 7 hours | 1.30 (1.03,1.57) | 0.020 | 1.27 (1.01,1.53) | 0.047 |  |
| > 8 hours | 1.36 (1.05,1.69) | 0.001 | 1.33 (1.06,1.86) | 0.001 |  |

Model was adjusted age, sex, marital status, PIR, educational level, obesity, smoke, alcohol use, sleep trouble, PA, sleep duration, and work schedule.

Table S4 Joint analysis of CUS and different sleep duration regarding the aging risk

|  | Workdays |  | Weekends |  |
| --- | --- | --- | --- | --- |
|  | OR (95%CI) | P | OR (95%CI) | P |
| Sleep for 7-8 h and with CUS | Ref. |  | Ref. |  |
| Sleep for 7-8 h and without CUS | 1.28 (0.91,1.80) | 0.106 | 1.15 (0.79,1.66) | 0.322 |
| Sleep less than 7 h and with CUS | 1.31 (0.86,1.98) | 0.132 | 1.26 (0.86,3.79) | 0.197 |
| Sleep less than 7 h and without CUS | 1.80 (0.96,3.43) | 0.063 | 1.52 (1.04,2.42) | 0.004 |
| Sleep more than 8 h and with CUS | 1.25 (0.72,2.17) | 0.294 | 0.99 (0.63,1.57) | 0.969 |
| Sleep more than 8 h and without CUS | 1.59 (1.13,2.23) | 0.023 | 1.63 (1.02,2.71) | 0.008 |

Model was adjusted age, sex, marital status, PIR, educational level, obesity, smoke, alcohol use, sleep trouble, PA, sleep duration, and work schedule.

Table S5 Subgroup analysis

| Without sleep disorder |  |  | With sleep disorder |  |
| --- | --- | --- | --- | --- |
| Weekend CUS |  | P |  | P |
| No | Ref. |  | Ref. |  |
| Yes | **0.75 (0.56, 1)** | **0.049** | 0.84 (0.59, 1.21) | 0.307 |
| Weekend CUS duration (h) |  |  |  |  |
| ≤0 | Ref. |  | Ref. |  |
| 0-1* | 0.72 (0.51, 1.01) | 0.056 | 0.74 (0.49, 1.14) | 0.124 |
| 1-2* | **0.69 (0.49, 0.96)** | **0.036** | 0.85 (0.40, 1.81) | 0.592 |
| >2 | 0.95 (0.53, 1.71) | 0.834 | 1.01 (0.43, 2.41) | 0.966 |
| Without OSA(Snore Rarely or Never) |  |  | OSA(Snore Frequently ) |  |
| Weekend CUS |  | P |  | P |
| No | Ref. |  | Ref. |  |
| Yes | 0.77 (0.57,0.96) | 0.007 | 0.83(0.61,1.19) | 0.336 |
| Weekend CUS duration (h) |  |  |  |  |
| ≤0 | Ref. |  | Ref. |  |
| 0-1* | 0.73 (0.53,1.05) | 0.196 | 0.77(0.53,1.39) | 0.429 |
| 1-2* | 0.67 (0.47,0.93) | 0.005 | 0.70 (0.33,1.12) | 0.434 |
| >2 | 0.96 (0.59,1.89) | 0.891 | 1.12(0.50,2.56) | 0.891 |
| Not retired |  |  | Retired |  |
| Weekend CUS |  | P |  | P |
| No | Ref. |  | Ref. |  |
| Yes | 0.82 (0.59, 1.15) | 0.178 | 1.12 (0.72, 1.75) | 0.532 |
| Weekend CUS duration (h) |  |  |  |  |
| ≤0 | Ref. |  | Ref. |  |
| 0-1* | 0.72 (0.51, 1.02) | 0.060 | 1.08 (0.53, 2.20) | 0.766 |
| 1-2* | **0.71 (0.51, 0.98)** | **0.046** | 1.64 (0.47, 5.74) | 0.299 |
| >2 | 1.03 (0.64, 1.66) | 0.878 | 0.69 (0.33, 1.41) | 0.197 |

*0h<Weekends CUS duration≤1h, 1h<Weekends CUS duration≤2h.

Model 1 was crude model.

Model 2 was adjusted age, sex, marital status, PIR, educational level, obesity

Model 3 was adjusted age, sex, marital status, PIR, educational level, obesity, smoke, alcohol use, sleep trouble, PA, sleep duration in work day.

Table S6 The relationship between weekend CUS and aging risk in different age groups

| Age<30 | OR (95% CI) | P |
| --- | --- | --- |
| Weekends CUS |  |  |
| No | Ref. |  |
| Yes | 0.63 (0.54, 0.74) | <0.001 |
| 30≤Age<40 |  |  |
| Weekends CUS |  |  |
| No | Ref. |  |
| Yes | 0.67 (0.53, 0.84) | 0.001 |
| 40≤Age<50 |  |  |
| Weekends CUS |  |  |
| No | Ref. |  |
| Yes | 0.78 (0.62, 0.97) | 0.030 |
| 50≤Age<60 |  |  |
| Weekends CUS |  |  |
| No | Ref. |  |
| Yes | 0.84 (0.63, 1.12) | 0.239 |
| Age>60 |  |  |
| Weekends CUS |  |  |
| No | Ref. |  |
| Yes | 0.81 (0.66, 1) | 0.051 |

Model was adjusted age, sex, marital status, PIR, educational level, obesity, smoke, alcohol use, sleep trouble, PA, sleep duration in work day.

Table S7 Sensitive analysis

| Sensitivity analysis 1^a^ | | | Sensitivity analysis 2^b^ | | Sensitivity analysis 3^c^ | | Sensitivity analysis 4^d^ | |
| --- | --- | --- | --- | --- | --- | --- | --- | --- |
| Weekend CUS | OR (95%CI) | P | OR (95%CI) | P | OR (95%CI) | P | OR (95%CI) | P |
| No | Ref. |  | Ref. |  | Ref. |  | Ref. |  |
| Yes | **0.77 (0.59, 0.98)** | **0.040** | **0.79 (0.62,1)** | **0.047** | **0.71 (0.55,0.91)** | **<0.001** | **0.76 (0.58,0.97)** | **0.037** |
| Weekend CUS duration (h) |  |  |  |  |  |  |  |  |
| ≤0 | Ref. |  | Ref. |  | Ref. |  | Ref. |  |
| 0-1* | **0.73 (0.55, 0.95)** | **0.029** | **0.76 (0.59,0.97)** | **0.035** | **0.74 (0.57,0.95)** | **0.002** | **0.73 (0.55,0.98)** | **0.039** |
| 1-2* | **0.69 (0.54, 0.88)** | **0.011** | **0.73 (0.57,0.93)** | **0.024** | **0.70 (0.54,0.90)** | **0.017** | **0.70 (0.54,0.90)** | **0.016** |
| >2 | 0.91 (0.57, 1.45) | 0.632 | 1 (0.60,1.67) | 0.999 | 0.94 (0.55,1.59) | 0.838 | 0.92 (0.55,1.54) | 0.700 |

*0h<Weekends CUS duration≤1h, 1h<Weekends CUS duration≤2h.

^a^Without sleep or psychiatric medication (N=4430), this model was adjusted age, sex, marital status, PIR, educational level, obesity, smoke, alcohol use, sleep trouble, PA, sleep duration in work day.

^b^Additional adjusted for work schedule(Only in participants who provided work schedule information, N=2650), this model was adjusted age, sex, marital status, PIR, educational level, obesity, smoke, alcohol use, sleep trouble, PA, sleep duration in work day, and work schedule.

^c^Excluded night shifts or participation without a work schedule provided (N=2338), this model was adjusted age, sex, marital status, PIR, educational level, obesity, smoke, alcohol use, sleep trouble, PA, sleep duration in work day, and work schedule.

^d^Exclude participants with CUS less than -30 minutes (N=4382), this model was adjusted age, sex, marital status, PIR, educational level, obesity, smoke, alcohol use, sleep trouble, PA, sleep duration in work day.
