# Supplementary material for: Relationship between weekends catch-up sleep and risk of aging
Source: PLoS One. 2025 Oct 8;20(10):e0332584. doi: 10.1371/journal.pone.0332584 (PMC12507208; doi:10.1371/journal.pone.0332584)
Supplement: Attachment — Submitted filename: response.docx [file pone.0332584.s005.docx]

Dear editor,

Thank you for your comments and recognition. We have noted that the reviewers have no further comments. Below is a point-by-point response to the editor's comments. We hope that all comments and issues can be addressed, making this manuscript more complete.

Additional Editor Comments:

Thank you for your thoughtful and thorough responses to the reviewers’ comments. The manuscript has significantly improved and is close to being ready for publication. To further strengthen the clarity, coherence, and overall readability of the paper, we encourage the authors to carefully review and consider the following editorial suggestions. These are intended to enhance the presentation and accessibility of your work for a broader readership. We look forward to receiving the revised version.
Abstract: In addition to stating the OR, the author may consider providing an interpretation (e.g., 20% lower odds [OR 0.8, 95% CI: 0.63–1]).
Response: Thank you for your comments; we have made corresponding revisions.
Intro: A more comprehensive literature review is recommended to better establish the rationale for the study, particularly regarding Weekend Catch-Up Sleep and the Risk of Aging.
Response: Thank you for your comments. There is indeed a scarcity of literature reviews on catch-up sleep and aging. We have replaced it with a more comprehensive literature review related to aging and catch-up sleep.
Exposure Assessment: I may have missed it, but I did not see the paper “[5] Kim et al. (2021),” which assessed the relationship between weekend CUS and other factors such as phenotypic age and anti-aging effects. Please clarify.
Response: Thank you for this comment. We sincerely apologize for the incorrect order of the literature citations due to issues with the EndNote software. This has now been corrected.
Statistical Analysis: The authors may consider explaining the rationale for adjusting for age when age itself is the outcome variable. If aging (phenotypic age) is used as the outcome, why was self-reported age included as an adjustment variable? Similarly, for other adjusted variables, how do they justify their inclusion in the model? For example, if education and BMI were not significantly associated, what was the rationale for including them?

Response: Thank you for this insightful comment. Regarding the inclusion of self-reported chronological age as an adjustment variable when phenotypic age (a measure of biological aging) is the outcome: Chronological age and phenotypic age, while correlated, represent distinct constructs—chronological age reflects the passage of time, whereas phenotypic age captures biological aging processes that may diverge from chronological age (e.g., individuals with the same chronological age can exhibit different rates of biological aging). By adjusting for chronological age, we aim to isolate the association between CUS and accelerated biological aging (i.e., phenotypic age relative to chronological age), rather than conflating it with the general aging process tied to time. This approach aligns with methodological precedents in aging research, where adjusting for chronological age helps disentangle biological aging from chronological progression.

For other adjusted variables (e.g., education, BMI), their inclusion was guided by theoretical and empirical evidence from prior literature, rather than solely by statistical significance in our dataset. Education is a well-established social determinant of health behaviors and aging trajectories, as it may influence health literacy, access to healthcare, and lifestyle factors (e.g., sleep patterns). BMI, as a marker of adiposity, has been consistently linked to biological aging processes, including inflammation and metabolic dysfunction, which are hypothesized to mediate aging risk. Even if these variables were not statistically significant in our analyses, their inclusion helps control for potential confounding—omitting them could introduce bias if they are associated with both CUS (exposure) and phenotypic age (outcome). This approach adheres to the principle of adjusting for a priori identified confounders to enhance the internal validity of our findings. We have supplemented the corresponding rationale in Supplementary Method 2 of the supplementary materials.

Results: Providing an interpretation of the results would enhance the paper’s readability.
Response: Thank you for your comment. We have added some interpretations and descriptions of the results part.

Conclusion: Is this a cohort study? The author previously described it as cross-sectional—please clarify.

Response: Thank you for your comments; we have made corresponding revisions.
Tables: Authors may consider formatting the mean and SD as Age (mean±sd); Could you please consider indicating which tests were undertaken for which variables in the table, may be by using the endnote? I mean which mean diff was t-test and which one is Mann-Whitney U tests; Indicating the sig. using '*' would be useful.

Response: Thank you for your comments; we have made corresponding revisions.

These are all our responses, and we hope they can address the above comments.

All the best.

Nan Yao.
