# Supplementary material for: Relationship between weekends catch-up sleep and risk of aging
Source: PLoS One. 2025 Oct 8;20(10):e0332584. doi: 10.1371/journal.pone.0332584 (PMC12507208; doi:10.1371/journal.pone.0332584)
Supplement: Attachment — Submitted filename: Reviewer comment.docx [file pone.0332584.s002.docx]

In this study, the authors investigate the relationship between weekend Catch-Up Sleep (CUS) and biological aging using data from NHANES 2017-2018. The study presents an interesting perspective on how sleep compensation on weekends may influence aging risk, particularly in individuals with different sleep habits and durations. While the findings are intriguing, several methodological and conceptual concerns need to be addressed to strengthen the manuscript.

**Major Concerns:**

Major Comment #1: Specification Regarding Sleep Disorders, specially Sleep Apnea

The manuscript considers sleep disorders in subgroup and sensitivity analyses but does not specify which disorders are included. Given that NHANES 2017-2018 contains specific questions on obstructive sleep apnea (OSA), snoring, and breathing interruptions during sleep, it is unclear whether these factors were considered. OSA is particularly relevant as it is linked to inflammation, metabolic dysfunction, and accelerated biological aging—factors that could confound the observed relationship between CUS and aging.

Additionally, while the manuscript states that sleep disorder information was obtained from NHANES, it appears to rely on the broad self-reported question about "trouble sleeping." This approach does not distinguish between insomnia, OSA, or restless legs syndrome, which may have differing impacts on aging.

The authors should clarify whether:

- OSA was included as a distinct category in the analysis. If not, this is a significant limitation that should be acknowledged.
- Snoring and breathing irregularities were considered as proxies for undiagnosed OSA.
- Additional subgroup analyses could be performed, distinguishing between different sleep disorder types.

Furthermore, the sensitivity analysis excludes participants taking sleep and psychiatric medications, but the manuscript does not specify which medications were excluded. Since benzodiazepines, non-benzodiazepine hypnotics, and antidepressants can alter sleep architecture and affect biological aging, this exclusion criterion should be explicitly defined.

Major Comment #2: Mediation and Moderation Analyses

The current statistical approach categorizes weekend CUS duration into discrete groups (e.g., no CUS, 0-1h, 1-2h, >2h), which may limit the ability to detect more nuanced relationships. A more robust approach would be to apply moderation and mediation analyses, which could provide deeper insight into the role of CUS in aging.

- Mediation Analysis: CUS duration could act as a mediator between weekday sleep patterns (e.g., total weekday sleep, bedtime) and aging risk. This would help determine whether the protective effects of early bedtime or sufficient weekday sleep are explained through CUS.
- Moderation Analysis: Rather than treating CUS as a fixed categorical variable, it could be modeled as a moderator of the relationship between weekday sleep duration and aging. This would clarify whether CUS can buffer the negative effects of insufficient weekday sleep.

Applying these approaches would allow for a more continuous and dynamic understanding of sleep patterns rather than relying on static categorical cutoffs. If such analyses are not feasible with the current dataset, the authors should discuss this limitation in the manuscript.

Major Comment #3: Age-Stratified Analysis

The manuscript investigates the relationship between weekend CUS and aging but does not explicitly differentiate between young and older adults, either biologically or chronologically. While the authors use phenotypic age as a biomarker for biological aging, they do not analyze whether the effects of CUS differ across different age groups. This omission is important because aging is a continuous process, and the impact of sleep compensation on biological age might differ between younger and older individuals.

For example, the manuscript notes that CUS prevalence peaks in the 30-40 age group before declining, yet no further stratification is conducted to examine whether CUS has a differential effect on aging risk in younger versus older adults. A subgroup analysis based on age deciles or biologically defined aging categories (e.g., younger adults vs. middle-aged vs. older adults) would strengthen the findings and provide greater clarity on how CUS interacts with aging processes at different life stages.

Major Comment #4: Consideration for Cognitive Aging

The manuscript focuses on biological aging but does not address whether weekend CUS is related to cognitive aging. While NHANES 2017-2018 does not include direct cognitive tests it does provide metabolic and inflammatory biomarkers that could serve as indirect markers of cognitive decline.

Given the well-established link between sleep, aging, and cognitive function, the authors should discuss whether their aging measure captures cognitive aspects or is limited to metabolic/physiological aging. If possible, additional analyses could examine how CUS relates to metabolic and inflammatory markers that are relevant for cognitive aging.

Major Comment #5: Limited Citations

The introduction primarily relies on single references per claim, which may limit the depth of evidence supporting its conclusions. While citing individual studies is valuable, certain claims—especially those regarding the effects of sleep and aging—would benefit from referencing multiple sources to provide a broader scientific foundation.

To strengthen the introduction, the authors should increase citation density by incorporating multiple studies for each major claim.
